# Anti-anxiety and hypnotic effects of ethanolic and aqueous extracts of *Lippia citriodora* leaves and verbascoside in mice

**Published:** 2017

**Authors:** Bibi Marjan Razavi, Naser Zargarani, Hossein Hosseinzadeh

**Affiliations:** 1 *Targeted Drug Delivery Research Center, Department of Pharmacodynamy and Toxicology, School of Pharmacy, Mashhad University of Medical Sciences, Mashhad, Iran*; 2 *School of Pharmacy, Mashhad University of Medical Sciences, Mashhad, Iran*; 3 *Pharmaceutical Research Center, Department of Pharmacodynamy and Toxicology, School of Pharmacy, Mashhad University of Medical Sciences, Mashhad, Iran*

**Keywords:** Lippia citriodora, Verbascoside, Anti-anxiety, Hypnotic, Elevated plus maze

## Abstract

**Objectives:**

The extract of *Lippia citriodora* and its main component, verbascoside, are known for their hypnotic effects in traditional medicine. In this study, the anxiolytic and hypnotic effects of *L. citriodora* leave extracts and verbascoside were evaluated in mice.

**Materials and Methods:**

Animals were divided into 11 groups of six mice each. Group I received normal saline, Group II received Diazepam (2 mg/kg) as positive control, Groups III, IV and V received *L. citriodora* ethanolic extracts (50, 100 and 200 mg/kg, respectively), Groups VI, VII and VIII received *L. citriodora *aqueous extracts (50, 100 and 200 mg/kg, respectively) and Groups IX, X and XI received Verbascoside (25, 50 and 100 mg/kg, respectively). All agents were administrated intraperitoneally. To evaluate hypnotic activity, pentobarbital sleeping test, and for anxiolytic activity, elevated plus-maze (EPM), locomotor activity, open field and motor coordination (rotarod test) tests were used. To understand the role of GABA_A_ receptor, flumazenil was also administered.

**Results:**

The extracts and verbascoside increased the time spent and number of entries in the open arms of EPM. Moreover, these agents significantly increased the sleeping time induced by pentobarbital. In addition, the highest dose of extracts and verbascoside reduced time spent on the rod and total locomotion in the open field tests, respectively. Flumazenil inhibited the effects of extracts and verbascoside in EPM and hypnotic tests.

**Conclusion:**

These results suggested that ethanolic and aqueous extracts of *L. citriodora* and verbascoside exhibit anxiolytic, hypnotic and muscle relaxant effects especially at the highest doses and these effects are partially due to the interaction with GABA_A_ receptor.

## Introduction

The genus *Lippia* (Verbenaceae) contains approximately 200 species of herbs, shrubs and small trees (Pascual et al., 2001 ▶). *Lippia citriodora* (lemon verbena), is one of the most important species of Verbenaceae. It is extensively distributed throughout South and Central America and tropical African countries. It also grows in European countries and Iran, but is not native to them (Pascual et al., 2001 ▶). This plant has been widely used in food, cosmetic and household products industries (Alavi et al., 2011 ▶). *L. citriodora* leaves are used in foods as flavoring agents. Different beneficial properties of *L. citriodora* have been reported both in traditional and modern medicines (Oskouei Shirvan et al., 2016 ▶). The leaves of this plant are utilized for the treatment of fever, neuropathic and stomach pains, dizziness, headaches, hypnotic, anemia, migraine, and cold symptoms (Pascaul et al., 2001 ▶). Furthermore, pharmacological studies indicated that *L. citriodora* leaves exhibit different properties including antioxidant (Lenoir et al., 2011 ▶), antinociceptive and anti-inflammatory (Mehrabizadeh Honarmand et al., 2011 ▶) and anti-bacterial (Koohsari et al., 2013 ▶) effects. 

Verbascoside, is a phenylpropanoid glycoside isolated from *L. citriodora* and several other medicinal plants. According to several lines of evidence, verbascoside has been reported to possess multiple beneficial effects including anti-inflammatory (Sanchez et al., 2013 ▶), anti-ulcerogenic (Sanchez et al., 2013 ▶), anti-bacterial (Pereira et al., 2014 ▶), anticancer (Zhang et al., 2014 ▶), antioxidant (Di Giancamillo et al., 2015 ▶), antithrombotic (Campo et al., 2015 ▶), immunomodulatory (Pastorelli et al., 2012 ▶), analgesic (Isacchi et al., 2011), cardioprotective (Campo et al. 2015 ▶), neuroprotective (Liang et al., 2016 ▶) and gastroprotective (Moura et al., 2015 ▶) effects. 

Recently, herbal medicines have been used for the treatment of a variety of diseases including neurobehavioral disorders because of their safety, efficacy, cultural acceptability and fewer side effects (Ernst, 2006 ▶). Hypnotic and anti-anxiety activities of some plants including *Echium italicum* L. (Hosseinzadeh et al., 2012 ▶), *Pistacia vera* (Ziaee and Hosseinzadeh 2010 ▶), *Crocus sativus* (Hosseinzadeh and Noraei 2009 ▶) and *Salvia leriifolia* Benth (Hosseinzadeh et al., 2008 ▶), and their active components have been evaluated in our previous studies. According to several investigations, *L. citriodora* and its main constituents exhibit potent neuroprotective effects including anti-parkinson and memory-enhancing activities through different mechanisms such as antioxidant, anti-inflammatory and antiapoptotic effects (Esposito et al., 2010 ▶; Gao et al., 2015 ▶; Liang et al., 2016 ▶; Amin et al., 2016 ▶). 

In recent studies, oxidative stress has been shown to be associated with neurobehavioral disorders such as anxiety and depression (Bouayed et al., 2009 ▶). So, because of the potential causal role of oxidative stress in anxiety, attention has been focused on a wide array of natural antioxidants such as *L. citriodora* and its main constituents, verbascoside for medicinal purposes. Considering the traditional use of this plant as a hypnotic and sedative agent, this study was done to evaluate the anxiolytic and hypnotic effects of the *L. citriodora* and its main constituent, verbascoside in several experimental models in mice.

## Materials and Methods

**Chemicals**

Verbascoside (Xian Aladdin Biological Technology), diazepam (Chimidaru Pharmaceutical Co., Iran) and normal saline (Daru pakhsh Pharmaceutical Co., Iran) were purchased. Flumazenil and sodium pentobarbital were obtained from Sigma.

**Animals**

Male mice weighing 20-30 g were obtained from a random bred colony in the animal house of Mashhad University of Medical Sciences. Mice were maintained in an environmentally controlled room (21±2°C) with a 12-hr light/12-hr dark cycle. Animals had free access to water and food. All animal experiments were carried out in accordance with Mashhad University of Medical Sciences, Ethics Committee Acts (Number of verification: 910172 ; the date of approval: 15.8.2012).

**Plant**

*L. citriodora* leaves were collected from the surrounding areas of Karaj city, Alborz province, Iran, dried in shadow and ground to powder. *L. citriodora* was identified by the Department of Botany in Ferdowsi University, Mashhad, Iran and voucher samples were kept for reference in the herbarium of the Department of Pharmacognosy, School of Pharmacy, Mashhad University of Medical Sciences, Iran (herbarium No. 12031). 

**Preparation of aqueous extract of** ***L. citriodora*** **leaves**

*L. citriodora *aqueous extract was prepared by adding 200 g of powdered plant material to 2 L of boiled water in a 2.5 L glass flask. Next, the mixture was heated for 15 min. The solution was subsequently filtered using Whatman No. 1 filter paper and then concentrated under vacuum at 46 °C using a rotary evaporator. The residues obtained (20 g) were stored in a freezer at −20 °C until use (Portmann et al., 2012 ▶). 

**Preparation of ethanolic extract of** ***L. citriodora*** **leaves**

*L. citriodora* ethanolic extract was prepared by macerating 200 g of powdered plant material to 1200 ml of absolute ethanol in a 2.5 L glass flask and the mixture was shaken for 48 hours. The solution was subsequently filtered using Whatman No. 1 filter paper and then the solvent was evaporated at 30°C. The leftover extract (22 g) was kept in a freezer at −20 °C until use.

**Animal treatment**

Mice were divided into 11 groups of six each. Group I: 10 mL/kg normal saline as vehicle; Group II: Diazepam (2 mg/kg) as positive control; Groups III, IV and V: *L. citriodora* ethanolic extracts at doses of 50, 100 and 200 mg/kg, respectively (Mehrabizadeh Honarmand et al., 2011 ▶); Groups VI, VII and VIII: *L. citriodora *aqueous extracts at doses of 50, 100 and 200 mg/kg (Mehrabizadeh Honarmand et al., 2011 ▶), respectively; Groups IX, X and XI: Verbascoside at doses of 25, 50 and 100 mg/kg (**Isacchi et al. 2011**). All agents were administrated intraperitoneally and dissolved in normal saline.

**Elevated Plus-Maze**

The elevated plus maze (EPM) was used to evaluate the anti-anxiety effects of *L. citriodora* and verbascoside. The apparatus consisted of two open arms and two closed arms and was located 50 cm above the floor. Mice received the aforementioned agents 30 min before the EPM test. Then, mice were placed at the center of the plus maze and the number of the entries into the open arms and the time spent on the open arms were recorded for a period of 5 min by Maze router Software (V3.1, Iran). The percentage of the entries into the open arms and the percentage of the time spent in the open arms were calculated by the following formula: 

Number of the entries into the open arms or the time spent in the open arms/ total number of the entries or total time spent in the open and closed arms × 100 (Fachinetto et al., 2007 ▶). To evaluate the role of GABA_A_ receptor in anxiolytic properties of *L. citriodora*, in another experiment, flumazenil (10 mg/kg), an antagonist of benzodiazepine (BZD) site in the GABA_A_-BZD receptor complex was administrated 15 min prior to the administration of the extracts and verbascoside (Hosseinzadeh and Sadeghnia, 2007 ▶). Afterwards, each animal was introduced to EPM. 

**Open field test**

The open field apparatus, made of white wood, had a floor of 100×100 cm divided by red lines into 25 squares of 20×20 cm. The walls (50 cm high) were also painted in white. The test room was illuminated at the same intensity of that of the colony room. Mice received the mentioned agents one hour before test. Each animal was placed in the center of the open field, and its behavior was observed for 5 min. The parameters evaluated were the total number of squares crossed, the number of outer squares (those adjacent to the walls) crossed and the number of inner squares crossed which were total, peripheral, and central locomotion, respectively. At the end of each test, the open field apparatus was cleaned with a wet sponge and a dry paper towel (Pardon et al., 2000 ▶).

**Pentobarbital sleeping time**

*L. citriodora* ethanolic and aqueous extracts as well as verbascoside were administered intraperitoneally 30 min before the test. The mice were considered asleep if stayed immobile and lost its righting reflex when positioned on its back. The time interval between pentobarbital injection and the onset of sleep was recorded as sleep latency. The time from the loss of rightness reflex to awakening (duration of sleeping) were also determined (in minutes) for each animal (Hossinzadeh and Norani, 2009 ▶). To evaluate the role of GABA_A_ receptor in hypnotic properties of *L. citriodora*, in another experiment, flumazenil (10 mg/kg), was administrated 15 minutes prior (Hosseinzadeh and Sadeghnia, 2007 ▶) to administration of the extracts and verbascoside. Afterwards, each animal was tested for pentobarbital sleeping time as described above.

**Rotarod test**

Motor coordination and balance were evaluated by the accelerating Rotarod (TSE RotaRod System, Germany). Mice were placed on a horizontal metal rod coated with rubber (3 cm diameter) rotating at an initial speed of 10 rpm/min. Final speed of the rod was 20 rpm in accelerated studies and the rotational velocity of the rod was increased linearly from 10 to 20 rpm within 20 sec. The time that each animal could maintain its balance walking on top of the rod was measured. Mice were given two trials with a maximum period of 300 sec and a 30-60 min inter-trial rest interval.

Before the beginning of all experiments, the riding ability of the animals on the Rotarod was checked. Thus, the mice were initially put on a rotating rod, and mice that immediately dropped off (within 30 se) were removed from the experiment (Hossinzadeh and Norani, 2009 ▶).

**HPLC fingerprinting of the aqueous and ethanolic extracts of** ***L. citriodora***

Semi-preparative HPLC–DAD was performed on a KNAUER liquid chromatograph system consisting of a quaternary pump (Smartline Pump 1000). Detection was carried out using UV–VIS diode array detector (Smartline DAD 2800), and data were processed using EZChrom Elite software. The aqueous and ethanolic extracts were subjected to reverse-phase HPLC using a gradient method of 20–100% methanol in water as the eluent including 0.05% trifluoroacetic acid. The preparative C18 (5µ, 21.2 × 250 mm) and a flow rate of 10 ml/min were used. The peaks were monitored at 320 nm.

**Statistical analysis**

Data are expressed as mean±SEM. Statistical analysis was performed using one-way ANOVA followed by the Tukey-Kramer *post-hoc* test for multiple comparisons. Values of p<0.05 were considered to be statistically significant.

## Results

**Elevated plus-maze**

**Figure 1:**
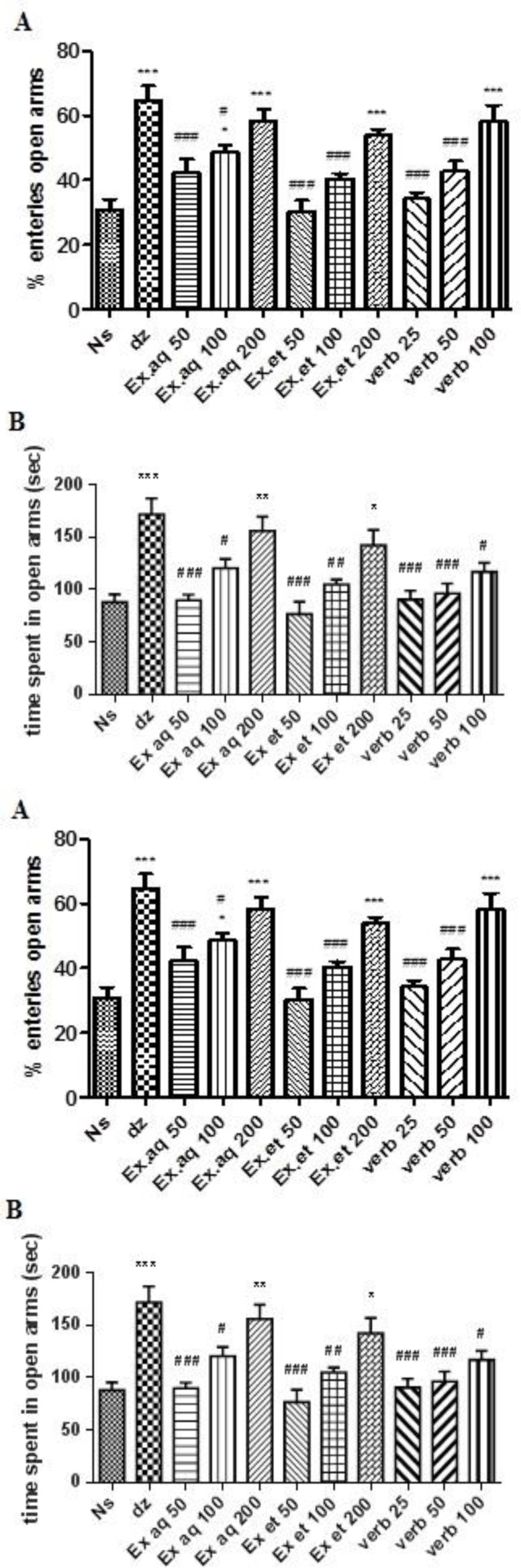
Effects of ethanolic and aqueous extracts of *L. citriodora *and verbascoside on the percentage of the open arm entries of the elevated plus-maze (EPM) (A) and on the time spent in open arm of the elevated plus-maze (EPM) (B). Effect of flumazenil on the percentage of entries into open arms (C) and on the percentage of time spent in open arms (D) by mice treated with ethanolic and aqueous extracts of *L. citriodora* and verbascoside in EPM test. Data are presented as mean±SEM of a group of six mice. *, ** and *** indicate p<0.05, p<0.01 and p<0.001, respectively, compared to normal saline. ^#^, ^##^ and ^###^ indicate p<0.05, p<0.01 and p<0.001, respectively, compared to diazepam. ^+^,^ ++ ^and^ +++^ indicate p<0.05, p<0.01 and p<0.001 respectively compared to group in the absence of flumazenil. Statistical analysis were performed using Tukey-Kramer test. Ns: normal saline; dz: diazepam; Ex aq: aqueous extract; Ex et: ethanolic extract; verb: verbascoside; flo: flumazenil

*L. citriodora* ethanolic (200 mg/kg, p<0.001) and aqueous (100 and 200 mg/kg, p<0.05 and p<0.001, respectively) extracts as well as verbascoside (100 mg/kg, p<0.001) caused a significant increase in the percentage of entries into the open arms of maze compared with normal saline (Figure 1A). As shown in Figure 1B, treatment with the *L. citriodora* ethanolic (200 mg/kg, p<0.05) and aqueous (200 mg/kg, p<0.01) extracts increased the time spent in the open arms compared with normal saline. Diazepam (2 mg/kg), as the positive control, increased the percentage of entries into the open arms and diazepam-treated mice spent more time in the open arms of the maze (Figures 1A and aB). The percentage of the entries into the open arms by mice treated with the highest doses of extracts and verbascoside (effective doses) showed that flumazenil significantly decreased this parameter in all groups. In addition, flumazenil decreased the time spent in open arms significantly in all groups at the highest doses (Figures 1C and 1D).

**Open field test**

**Figure 2:**
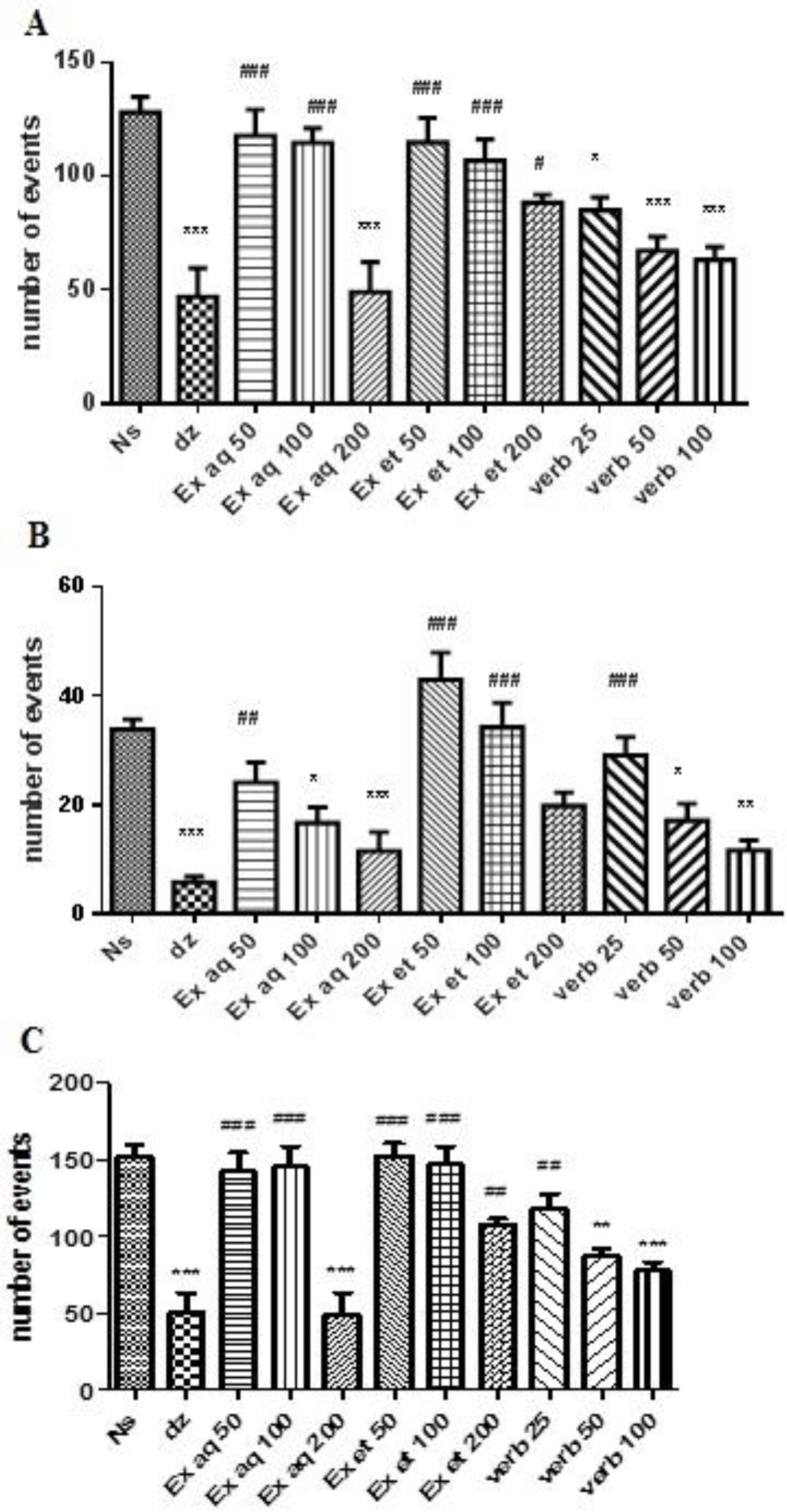
Effects of ethanolic and aqueous extracts of *L. citriodora *and verbascoside on the peripheral (A), central (B) and total (C) locomotion in open field test. Data are presented as mean ± SEM of a group of six mice. *, ** and *** indicate p<0.05, p<0.01 and p<0.001, respectively, compared to normal saline. ^#^, ^##^ and ### indicate p<0.05, p<0.01 and p<0.001, respectively, compared to diazepam. Statistical analysis were performed using Tukey-Kramer test. Ns: normal saline; dz: diazepam; Ex aq: aqueous extract; Ex et: ethanolic extract; verb: verbascoside

The aqueous extract at dose of 200 mg/kg (p<0.001) and verbascoside at three doses (p<0.05 and p<0.001) decreased peripheral locomotion as compared with normal saline (Figure 2A). Results also showed that aqueous extract (100 and 200 mg/kg, p<0.05 and p<0.001, respectively) and verbascoside (50 and 100 mg/kg, p<0.05 and p<0.01, respectively) decreased peripheral locomotion (Figure 2B). 

A reduction in total locomotion was observed in groups which received aqueous extract at the dose of 200 mg/kg (p<0.001) and verbascoside at doses of 50 and 100 mg/kg (p<0.01 and p<0.001, respectively) (Figure 2C). Diazepam decreased the open field parameters (Figure 2). 

**Rotarod test**

**Figure 3:**
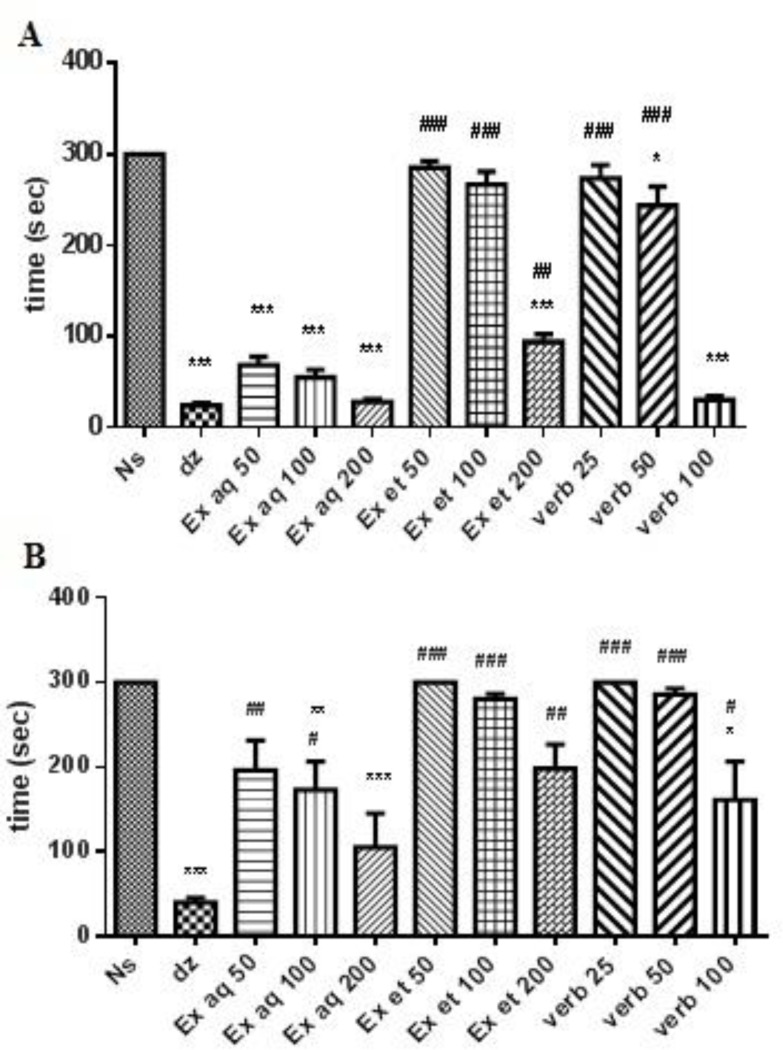
Effects of ethanolic and aqueous extracts of *L. citriodora* and verbascoside on motor coordination in Rotarod test, 30 (A) and 60 min (B) after the IP administration of the extracts or diazapam. Data are presented as mean ± SEM of a group of six mice.*, ** and *** indicate p<0.05, p<0.01 and p<0.001, respectively, compared to normal saline. ^#^, ^##^ and ^###^ indicate p<0.05, p<0.01 and p<0.001, compared to diazepam. Tukey-Kramer test. Ns: normal saline; dz: diazepam; Ex aq: aqueous extract; Ex et: ethanolic extract; verb: verbascoside

The aqueous extract at three doses, ethanolic extract at the highest dose and verbascoside at doses of 50 and 100 mg/kg significantly decreased motion balance and function in the Rotarod system at 30 min after injection (Figure 3A), whereas, the aqueous extract (100 and 200 mg/kg) and verbascoside (100 mg/kg) reduced motor coordination at 60 min after injection, significantly (Figure. 3B). Diazepam decreased this parameter compared with the normal saline (Figure 3).

**Pentobarbital sleeping time**

**Figure 4:**
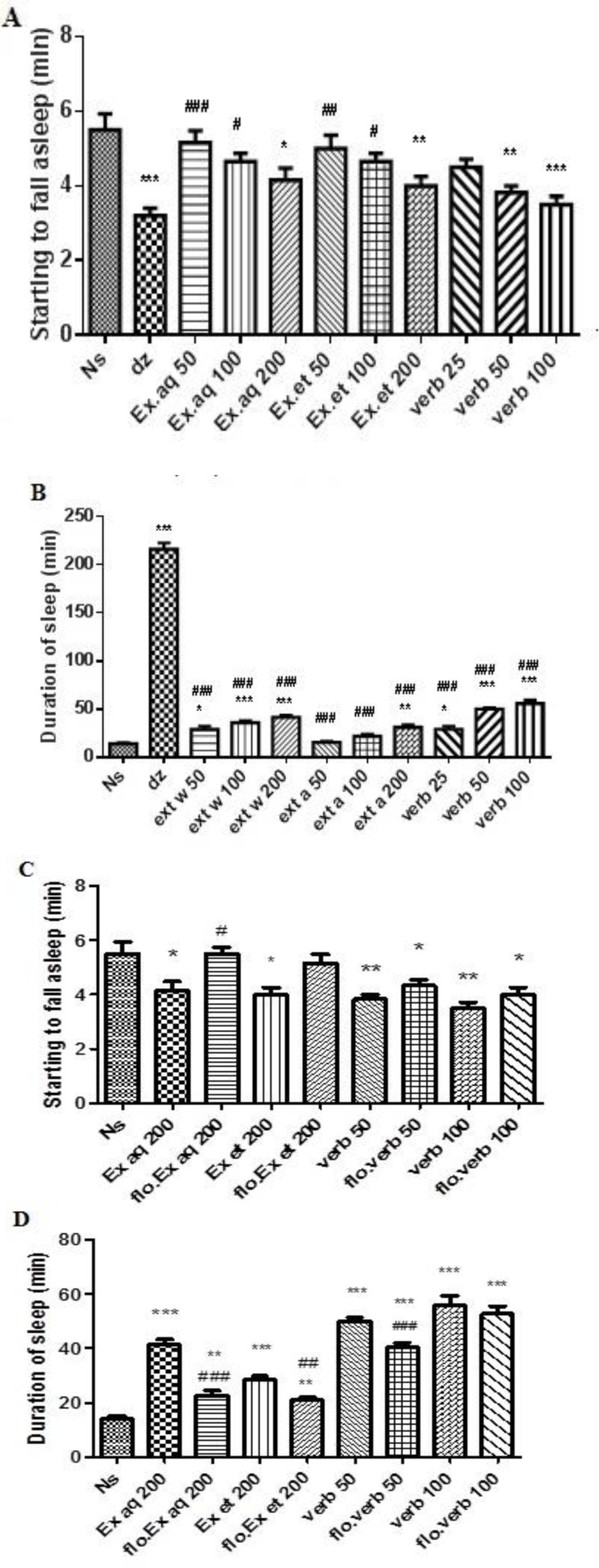
Effects of ethanolic and aqueous extracts of *L. citriodora* and verbascoside on sleep latency (A) and sleeping time (B) on the potentiation of pentobarbital sleep test. Effect of flumazenil on sleep latency (C) and sleeping time (D) induced by ethanolic and aqueous extracts of *L. citriodora* and verbascoside in the potentiation of pentobarbital sleep test. Data are presented as mean ± SEM of a group of six mice.*, ** and *** indicate p<0.05, p<0.01 and p<0.001, respectively, compared to normal saline. ^#^,^ ##^ and ^###^ indicate p<0.05, p<0.01 and p<0.001, respectively, compared to diazepam. ^+^,^ ++ ^and^ +++^ indicate p<0.05, p<0.01 and p<0.001, respectively, compared to group in the absence of flumazenil. Statistical analysis were performed using Tukey-Kramer test. Ns: normal saline; dz: diazepam; Ex aq: aqueous extract; Ex et: ethanolic extract; verb: verbascoside; flo: flumazenil

The aqueous and ethanolic extracts (200 mg/kg) and verbascoside (50 and 100 mg/kg) decreased sleep latency significantly (Figure 4A). Moreover, three doses of aqueous extract, the highest dose of ethanolic extract and three doses of verbascoside could significantly increase sleeping time compared to normal saline (Figure 4B). Diazepam decreased the sleep latency and increased sleeping time (Figure 4). Results of the effect of flumazenil on sleep latency following treatment with the highest doses of extracts and verbascoside (effective doses), showed that flumazenil increased the sleep latency. This increase was significant in aqueous extract-treated mice (p<0.05). In addition, flumazenil significantly reduced sleeping time in aqueous and ethanolic extracts-treated mice (p<0.001 and p<0.01, respectively) (Figures 4C and 4D).

**The HPLC fingerprints**

**Figure 5:**
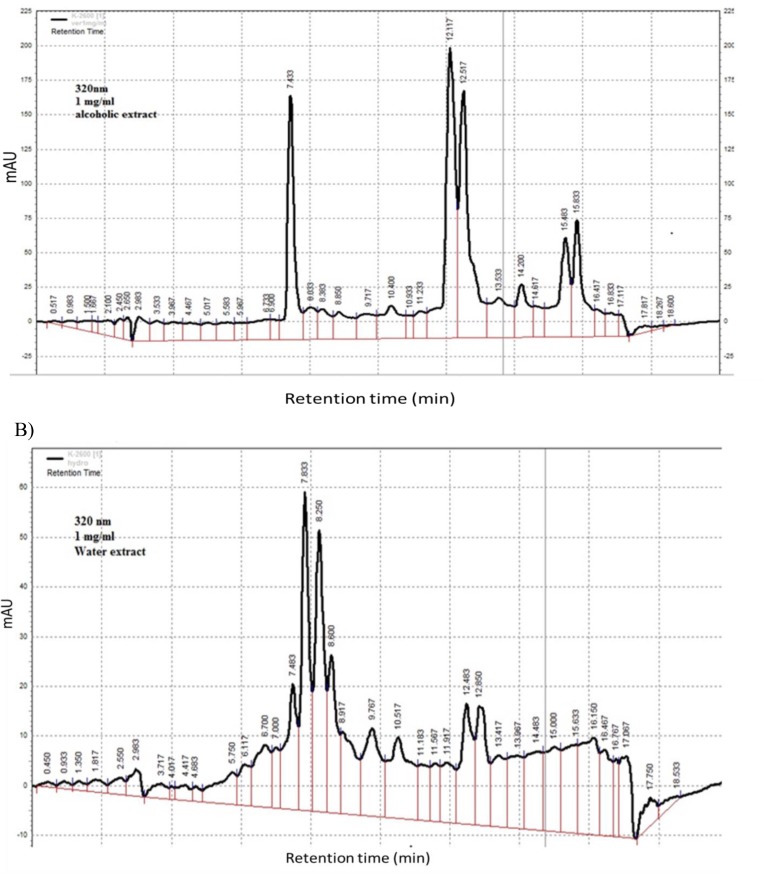
The HPLC fingerprints of the ethanolic (A) and aqueous (B) extracts of *L. citriodora*

The HPLC fingerprints of the ethanolic and aqueous extracts of *L. citriodora* showed major peaks at the retention times (min.) of 7.43, 12.11, 12.51, 14.2 , 15.48, 15.83 and 7.48, 7.83, 8.25, 8.60, 9.76, 10.51, 12.48, 12.85 at wavelength of 320 nm (Figure. 5).

## Discussion

*L. citriodora *leaves have been reported to possess sedative and hypnotic effects in traditional medicine (Yousefzadeh and Meshkatalsadat 2013 ▶.). Drugs currently used as sedative and anti-anxiety have some adverse effects including tolerance and dependence (Greenblattab and Shader 1978 ▶). In this study, anxiolytic and hypnotic effects of the *L. citriodora* aqueous and ethanolic leaves extracts and its main constituent, verbascoside, in several experimental models were evaluated in mice. Results showed that aqueous and ethanolic extracts of *L. citriodora* and verbascoside exhibit anti-anxiety, hypnotic and muscle relaxant activity. These effects were more obvious at the highest doses of extracts and verbascoside. In addition, according to the results, it might be concluded that hypnotic and anxiolytic activities of this plant are partly attributed to the interaction with GABA_A_ receptor. 

The elevated plus maze (EPM) is a commonly used behavioral assay for rodents and it has been validated for investigation of anti-anxiety effects of chemicals (Walf and Frye 2007 ▶). In this test, anxiolytic compounds decrease the natural aversion of animals to the open arms. Therefore, increases in the number of entry or the time spent in the open arms, reflect the anxiolytic effect of a compound (Hossinzadeh and Norani 2009 ▶). Our results showed that *L. citriodora* aqueous and ethanolic leaves extracts and verbascoside increased the entries into the open arms of EPM and augmented the time spent in the open arms, dose dependently. The anxiolytic activity of these extracts and verbascoside at the highest dose was similar to the effect of diazepam as a positive control. Our results are in the agreement with the findings of another study that showed that *L. citriodora* hydro-ethanolic leaves extract at the dose of 200 mg/kg increased the time spent in the open arms in EPM in rats (Eidi et al., 2014 ▶). In contrast to our results, another study indicated that administration of lemon verbena aqueous extract (10 - 1,000 mg/kg) increased anxiety‑like behaviors in EPM in rats (Veisi et al., 2016 ▶). Moreover, although the ethanolic extract of *L. citriodora* (100-800 mg/kg) did not show hypnotic activity in NMRI mice in one study (Bozorgmehr et al., 2012 ▶), our results revealed that the aqueous and ethanolic extracts as well as verbascoside decreased the sleep latency and significantly increased sleeping time as compared to normal saline in the potentiation of pentobarbital sleep test. This may be due to differences in mice strains and specific doses used in our study.

Several neurotransmitters have been implicated in the pathophysiology of anxiety disorders including dopamine, serotonin, glutamate and GABA (Nikolaus et al., 2010 ▶). Several studies done in animal models, molecular and clinical psychopharmacology, have proved that GABA_A_ receptor plays a pivotal role in the modulation of anxiety disorder (Liberzon et al., 2003 ▶). Diazepam, a benzodiazepine sedative-hypnotic drug, interacts with GABA_A_ receptor and possesses anti-anxiety, muscle relaxant and hypnotic effects. It seems that GABA_A_ receptor is involved in the anxiolytic and sedative effects of *L. citriodora* leave extracts as well as verbascoside because of the similarity of the effects of this plant to the effects of diazepam in different experimental models used in this study. So, to evaluate the role of GABA_A_ receptor in anxiolytic and hypnotic properties of *L. citriodora*, flumazenil (10 mg/kg), was administrated 15 minutes prior (Hosseinzadeh and Sadeghnia 2007 ▶) to administration of effective doses of extracts and verbascoside. Results showed that flumazenil significantly decreased the percentage of entries into the open arms and also reduced the time spent in the open arms of EPM in all groups. Furthermore, flumazenil could significantly reduce the sleeping time in aqueous and ethanolic extracts-treated mice in the potentiation of pentobarbital sleep test. Therefore, it might be concluded that the anti-anxiety effects of both extracts and verbascoside may be partly due to the interaction with GABA_A_ receptor. Results showed that flumazenil could significantly reduce the sleeping time in aqueous and ethanolic extracts in the potentiation of pentobarbital sleep test. Although the reduction of the sleeping time by flumazenil was observed in verbascoside-treated mice, but this effect was not statistically significant. This might be related to the limited number of the animals.

Phytochemical studies on *L. citriodora* leaves showed the presence of flavonoids in both aqueous and ethanolic extracts. The most commonly reported flavonoids were verbascoside, salvigenin, eupatorin, eupafolin, luteolin, hispidulin, diosmetin, cismaritin, cirsiliol, pectolin-arigenin and 6-hydroxyluteolin (Skaltsa and Shammas 1988 ▶).

Flavonoids are natural active components that tend to bind to benzodiazepine GABA_A_ receptors. Pharmacologically, they act as partial agonists. It has been reported that some semi-synthetic

Flavone derivatives are much more potent than diazepam *in vivo* (Wolfman et al., 1996 ▶). Baicalin, a flavonoid isolated from *Scutellaria lateriflora* L., was found to have anxiolytic activity which could be antagonized by a GABA_A_-specific antagonist (Kuroda et al., 2012 ▶). Chyrsin, another natural flavonoid isolated from *Passiflora coerulea *(Feliu-hemmelmann et al., 2013 ▶), and Wogonin, a flavonoid extracted from *Scutellaria baicalensis* Georgi (Hui et al., 2002 ▶) have been reported to exert anxiolytic activities. Therefore, anti-anxiety and hypnotic effects of this plant could be related to the presence of some flavonoids such as verbascoside. Our results showed that verbascoside, a phenylpropanoid compound, exhibits potent anti-anxiety, hypnotic and muscle relaxant activity. Verbascoside, the most abundant phenylpropanoid (97%) found in *L. citriodora* leaves, possesses several beneficial effects including antioxidant and anti-inflammatory effects.

 It has been shown that verbascoside (10 mg/kg) prolonged the time to exhaustion in treadmill exercise through inhibition of exercise-induced synthesis of 5-HT (5-hydroxytryptamine) and TPH2 (tryptophan hydroxylase) protein expression, and the increase of the 5-HT_1B_ protein level in the caudate putamen of Sprague-Dawley male rats (Zhou et al., 2016 ▶). Furthermore, inhibitory effect of verbascoside on neuronal nitric oxide synthase expression in lipopolysaccharide/interferon-gamma induced inflammation in glioma cells has been reported. Moreover, verbascoside reduced the expression of proinflammatory enzymes through inhibition of activation of nuclear factor kappa B and mitogen-activated protein kinase signaling pathway (Esposito et al. 2010 ▶). According to the results of another study, verbascoside protected against glutamate- induced neurotoxicity in primary cultured rat cortical cells through inhibition of glutamate-induced intracellular Ca^2+^ influx, suppression of overproduction of nitric oxide and reduction of reactive oxygen species formation. Verbascoside also restored the mitochondrial membrane potential and the activities of antioxidative enzymes (Koo et al., 2006 ▶).

According to the literature, nitric oxide synthase inhibitors could potentiate morphine anti-anxiety effects (Shin et al., 2003 ▶). Taken together, besides the involvement of some neurotransmitters such as GABA, oxidative stress, inflammation and increased nitric oxide production are also involved in the pathophysiology of anxiety disorders.

There are different mechanism of actions for muscle relaxation and hypnotic effects. Some medicines such as zolpidem has hypnotic effect with no relaxation effect or some drugs such as dantrolene causes skeletal muscle relaxation in animals without having marked CNS effects.

According to documents, the antioxidant activity of ethanolic leaf extract of *L. citriodora* is more than aqueous extract that may be due to the presence of higher amounts of phenolic compounds (El-Hawary et al. 2012 ▶). However, in this study, both ethanolic and aqueous extracts of *L. citriodora* as well as verbascoside exhibit similar anti-anxiety and hypnotic effects. This may be due to the presence of active components in both extracts (Figure 5). It has been shown that verbascoside can be immediately metabolized to hydroxytyrosol *in vivo* and hydroxytyrosol could enter the brain (Wu et al. 2009 ▶ ). Further studies are required to isolate the phenolic composition of both extracts to elucidate the exact mechanism of action. Taken together, *L. citriodora* leaves extracts and verbascoside, exhibit hypnotic and anxiolytic activities probably through different mechanisms including blockade of GABA_A_ receptor, antioxidant, anti-inflammatory and inhibition of nitric oxide production.

These results suggest that both ethanolic and aqueous extracts of *L. citriodora *and verbascoside exhibit anxiolytic, hypnotic and muscle relaxant effects especially at the highest dose. The mechanism of hypnotic and anxiolytic effects of this plant may be in part due to the interaction with GABA_A_ receptors. 
